# Dimensionality of the Center for Epidemiologic Studies Depression Scale: an exploratory bi-factor analytic study

**DOI:** 10.1007/s11136-015-1105-5

**Published:** 2015-08-18

**Authors:** Ted C. T. Fong, Cecilia L. W. Chan, Rainbow T. H. Ho, Jessie S. M. Chan, Celia H. Y. Chan, S. M. Ng

**Affiliations:** Centre on Behavioral Health, The University of Hong Kong, Pokfulam, Hong Kong; Department of Social Work and Social Administration, Jockey Club Tower, The Centennial Campus, The University of Hong Kong, Pokfulam, Hong Kong

**Keywords:** CES-D, Depression, General factor, Bi-factor model, Factor structure

## Abstract

**Objective:**

The Center for Epidemiologic Studies Depression Scale (CES-D) is a widely used instrument for measuring depressive symptoms. Though conventional factor analytic evaluations supported the use of four sub-scales for the CES-D, existing studies have yet to adopt the bi-factor analytic approach in psychometric assessment of the 20-item inventory. The present study aimed to apply both confirmatory factor analysis and exploratory bi-factor analysis to evaluate the dimensionality of the CES-D.

**Methods:**

Current scoring practice of the CES-D (single-factor, four-factor, and second-order models) was tested using confirmatory factor analyses in a sample of 706 Chinese persons with insomnia and depressive symptoms. As an alternative, exploratory bi-factor analysis was conducted to examine the utility of the general depression factor and specific factors.

**Results:**

Existing measurement models on the CES-D did not provide an adequate model fit to the data in terms of model fit indices and discriminant validity. The bi-factor model revealed a general depression factor that accounted for the majority of the item variance. The three specific factors (somatic symptoms, positive affect, and interpersonal problems) provided little unique information over and above the general factor and plausibly represent a methodological artifact rather than a substantive factor.

**Conclusion:**

The present study demonstrated empirical support for the bi-factor model as a realistic representation of the underlying structure of the CES-D. Researchers and clinicians are better served by simply using a single measure of depression.

## Introduction

Depression is one of the most prevalent mental health problems among metropolitan citizens. The Center for Epidemiologic Studies Depression Scale (CES-D) is one of the most commonly adopted self-report instruments for measuring the frequency of depressive symptoms [1]. The CES-D inquires about the frequency of 20 depressive symptoms during the week prior to measurement. Validation studies have shown adequate psychometric properties for the scale in terms of reliability and convergent validity in various populations in different countries such as depressive patients [2], community adults [3], college students [4], elderly primary care patients [5], and dementia caregivers [6]. The original developer of the CES-D [1] extracted four factors based on a principal component analysis and labeled them as depressed affect (seven items), somatic symptoms (seven items), positive affect (four items), and interpersonal problems (two items).

Though validation studies of the CES-D have in general revealed superior fit for the four-factor model than other measurement models in confirmatory factor analysis (CFA) [2–6], several methodological concerns should be noted regarding the four-factor model. First, previous studies applied principal component analysis and varimax rotation. Principal component analysis is known to be a biased estimator in factor analysis, and the orthogonal factors may likely lead to distorted factor structures [7, 8]. The eigenvalue >1 criterion is known to be unreliable and could lead to over-extraction of factors. Second, the factors of depressed affect and somatic symptoms were highly correlated (*r* = .86–.97) in the studies [2, 3, 6, 9]. The overly strong correlation casts doubts on the discriminant validity of the factors and signifies potential model redundancy. Third, the positive affect factor, which comprises solely four positively worded items, is plausibly a method factor that merely accounts for the wording effects [10]. Edwards and colleagues [11] found that a unidimensional model with a general depression factor and a method factor for those four items fit almost as well as the four-factor model. Fourth, the interpersonal problems factor is composed of only two items. It is in general not desirable to define factors by two indicators alone. Finally, there is the issue of making genuine cross-national comparisons and translation of the CES-D, with relatively few studies [5, 12] assessing the cross-ethnic measurement invariance of the CES-D.

As depression is a substantively complex and conceptually broad construct, the CES-D includes multiple indicators with diverse contents to assess various aspects of the construct (such as somatic complaints, negative mood, social withdrawal, and poor cognitive functioning). Nevertheless, researchers are most keenly interested in evaluating individuals on the general construct of depression. Because of the widespread use of the CES-D total score as a screening measure of depressive symptoms in clinical practice and research [13–15], it is important to uncover the precise dimensionality of the scale and explore the robustness of a unidimensional model.

The bi-factor model is an alternative and useful complement to traditional dimensionality analyses [16]. In a bi-factor representation, each item loads on a general factor that is assumed to underlie the items and explain their inter-correlations [17]. In addition, each item can load on none or one specific factor. The specific factors capture the item covariation that is independent of the general factor and provide unique information on specific domains over and above the general factor. In a bi-factor model, the general and specific factors are orthogonal to each other. Chen and colleagues [18] described the relative advantages of a bi-factor model over a second-order factor model. Bi-factor modeling can address a key question in dimensionality assessment, namely how much of the item variance is due to the general factor versus how much is due to secondary dimensions?

To our knowledge, bi-factor modeling has yet to be applied to previous psychometric studies of the CES-D. The purpose of the present study was to investigate the dimensionality of the CES-D in assessing depressive symptoms. Firstly, a number of existing measurement models of the CES-D––the single-factor model, the original four-factor model, and the second-order factor model––were evaluated and compared via CFA. Then, we proceed to evaluate the exploratory bi-factor model of the CES-D items. The use of a bi-factor analysis allowed us to empirically examine the usefulness of forming subscales, which would be clinically relevant to an evaluation of whether the CES-D factors offer an incremental value beyond the general depression factor.

## Methods

### Participants

This study was based upon a secondary data analysis of 706 Chinese persons with insomnia and depressive symptoms. The convenience sample was recruited in October 2013 via a clinical trial of qigong and body–mind–spirit interventions for emotional distress and sleep problems. The participants provided informed consent and completed an online questionnaire at home, in which the purpose, procedures, and potential risks of the study were clearly stated. The questionnaire took approximately 20 min to complete and included the CES-D and other self-report measures on anxiety, health-related quality of life, and sleep disturbance. Only baseline data were used in the present analyses. All of the procedures were approved by the institutional review board of the University of Hong Kong. The majority of the respondents were female (75.9 %) with a mean age of 51.0 years [standard deviation (SD) = 11.7]. Over half of the sample were married (62.6 %) and had tertiary education level (50.6 %). Of the 706 responses, 704 (99.7 %) provided complete data on all CES-D items.

### Measures

Depressive symptoms were assessed using the 20-item CES-D [1], which inquires about the frequency of depressive symptoms during the past week. The wordings of the CES-D items are given in Table 1. The response options consist of 4-point ordinal ratings coded as 0 (rarely or none of the time––less than 1 day per week), 1 (some or a little of the time––1–2 days per week), 2 (occasionally or a moderate amount of the time––3–4 days per week), and 3 (most or almost all of the time––5–7 days per week). The four items on positive affect were reverse-scored before computing a total score for the CES-D, with higher scores denoting greater depression.

**Table 1:** CES-D items and factor loadings for the bi-factor model with three specific factors

Item	Mean (SD)	General factor	Somatic symptoms	Positive affect	Interpersonal problems
1. Bothered by things	1.99 (0.99)	.72			
2. Poor appetite	0.96 (0.99)	.59			
3. Cannot shake blues	1.61 (1.13)	.88			
6. Depressed	1.51 (1.09)	.92			
9. Life was failure	1.66 (1.02)	.83			
10. Fearful	1.68 (1.08)	.83			
11. Restless sleep	1.69 (1.03)	.43			
13. Talked less than usual	1.69 (1.05)	.63			
14. Lonely	1.42 (1.12)	.83			
17. Crying spells	1.55 (1.11)	.61			
18. Sad	2.50 (0.74)	.89			
5. Trouble focusing	1.74 (0.98)	.84	.38		
7. Everything was effort	1.52 (1.03)	.83	.27		
20. Could not get going	1.64 (1.06)	.85	.35		
4. As good as others	1.01 (0.94)	.49		.44	
8. Hopeful about future	1.51 (1.04)	.62		.57	
12. Happy	0.82 (0.93)	.71		.51	
16. Enjoyed life	1.32 (1.04)	.68		.52	
15. People were unfriendly	1.07 (1.00)	.70			.49
19. Disliked by people	1.63 (1.06)	.70			.54
% of total variance explained		55	3	6	3

Only factor loadings that are greater than .20 are shown

The original authors of the CES-D proposed that a cutoff score of 16 or more was suggestive of clinically significant depression [1], and a higher cutoff point of 21 has been proposed for older individuals [15]. In the present study, the CES-D had a good Cronbach’s α [19] of .94 for the total score. The average total CES-D score was 30.5 (SD = 14.4). The total score did not differ significantly across the genders (female mean = 30.9, SD = 14.2 vs. male mean = 29.4, SD = 15.0; *p* > .05). Overall, 80.5 % of the respondents had total scores of 16 or more on the CES-D, and 71.2 % had scores of 21 or more. In the present study, the participants showed moderate to high level of depressive symptoms.

### Data analysis

In the present study, evaluation of the dimensionality of the CES-D was conducted in three steps. First, we performed CFA based on conventional approaches to estimate three existing measurement models, namely, the unidimensional model, original four-factor model, and second-order factor model using Mplus version 7.11 [20]. The single-factor model specifies that all of the 20 items are indicators of a single depression factor. In the four-factor model, the 20 items are assumed to measure four factors: depressed affect (seven items), somatic symptoms (seven items), positive affect (four items), and interpersonal problems (two items). For the second-order model, the four first-order factors load on a second-order depression factor.

Second, we performed exploratory bi-factor analyses for the CES-D [16, 17, 21] under BI-Geomin orthogonal rotation [22, 23]. A series of bi-factor analyses were specified for the ordinal items with a single general factor and up to three specific factors. Under the orthogonal rotation, the specific factors were uncorrelated with the general factor and other specific factors. The uncorrelated latent variables allowed the decomposition of the item variance to obtain the proportion of total variance explained by the general factor and the specific factors in an unequivocal way. Factor loadings that were statistically significant and greater than .40 in magnitude were taken to be practically significant [8].

All measurement models were estimated using the robust weighted least square estimator [24], which provides asymptotically unbiased and consistent parameter estimates and an accurate Chi-square test of fit [25] for the four-point ordinal CES-D items. Goodness of fit of the models was assessed based on the Chi-square (*χ*^2^) test and the model fit indices, namely comparative fit index (CFI), Tucker–Lewis index (TLI), root mean square error of approximation (RMSEA), and weighted root mean square residual (WRMR). The following criteria were used to evaluate the model fit indices [26, 27]: CFI ≥ .95; TLI ≥ .95; RMSEA ≤ .05; and WRMR ≤ .90. Because the Bayesian information criterion was not available with categorical indicators, model comparison was performed for nested models using the Chi-square difference test with the DIFFTEST option in Mplus [28].

Finally, we performed a multiple-indicator multiple-cause (MIMIC) analysis [29] based on gender and age. The MIMIC analysis examined potential gender and age biases in item responses and differences in latent variable means across age and gender [30]. Item biases across subgroups occurred where the items behaved differently for subgroups despite the same level of the latent variable. The MIMIC analysis was useful in assessing measurement invariance and comparability of the CES-D across different gender and age subgroups. Substantive direct effects were added from gender and age to the items to take into account the issue of differential item functioning. The effects of each covariate on the latent variable were displayed in SD units.

## Results

### Confirmatory factor models

Table 2 presents the fit indices of the three CFA models for the CES-D. The single-factor CFA model fits the data poorly with both CFI and TLI < .95, RMSEA > .10, and WRMR > .90. The original four-factor CFA model provided a marginal fit to the data. Although the factor indicators appeared to measure the four factors quite well with substantial loadings (*λ* > .40), the four factors were strongly correlated (*r* = .66–.94). The strong correlation (*r* = .94) between depressed affect and somatic symptoms implies potential model redundancy and casts doubts on the discriminant validity of the two factors. The second-order CFA model, which attempts to model the strong correlations among the four first-order factors by loading them on a higher-order factor, was a significantly poor fit to the data, compared with the original model (Δ*χ*^2^ = 9.8, Δ*df* *=* 2, *p* < .01*)*. The estimation of this second-order model resulted in a negative residual variance for the depressed affect factor with its factor loading on the second-order factor exceeding one. The Heywood case renders this model uninterpretable and may reflect model misspecification [31].

**Table 2:** Fit indices of the CFA models and bi-factor EFA models for the CESD

	*χ* ^2^	*df*	#	CFI	TLI	RMSEA	WRMR	Δ*χ* ^2^ (Δ*df)*
CFA model								
Single factor	2140.6	170	80	.933	.926	.128	2.325	/
Original four factor	795.1	164	86	.979	.975	.074	1.297	/
Four factor + second order	793.4	166	84	.979	.976	.073	1.313	9.8** (2)
Bi-factor EFA model								
1 general + 1 specific	1120.2	151	99	.967	.959	.095	1.460	
1 general + 2 specific	710.0	133	117	.981	.972	.078	1.048	−326.6** (18)
1 general + 3 specific	411.2	116	134	.990	.984	.060	.737	−238.4** (17)

*df* degree of freedom, # number of parameters, *CFI* comparative fit index, *TLI* Tucker–Lewis index, *RMSEA* root mean square error of approximation, *WRMR* weighted root mean square residuals, Δ*χ* ^2^ Chi-square difference test computed via DIFFTEST procedure for nested comparison with previous model, ** *p* < .01

### Exploratory bi-factor models

Table 2 displays the goodness-of-fit indices for the bi-factor CFA models with a general factor and up to three specific factors. The first five eigenvalues for the sample polychoric matrix were 11.4, 1.5, 0.9, 0.8, and 0.7, indicating that the ratio of the first to second eigenvalues was 7.4. The bi-factor models with one or two specific factors provided significant improvement in model fit over the unidimensional model in terms of the Chi-square difference test. However, the two models did not provide a satisfactory fit to the data. The bi-factor model with three specific factors showed adequate model fit indices and fits the observed data significantly better than any of the previous models.

Table 1 presents the factor loadings for the exploratory bi-factor model with one general factor and three specific factors. The item loadings on the general factor were statistically significant and substantial, with a range of .43 (restless sleep) to .92 (depressed) and an average *λ* = .73. The first specific factor was weakly measured by item 5 (trouble focusing), item 7 (everything was effort), and item 20 (could not get going) and resembled the somatic symptoms factor. The second factor was linked to the four positively worded items (item 4, item 8, item 12, and item 16) and corresponded to the positive affect factor. The third factor was measured by item 15 (people were unfriendly) and item 19 (disliked by people) and denoted the interpersonal problems factor. The general factor and specific factors accounted for 55, 3, 6, and 3 % of the total item variance, respectively. Of the 20 CES-D items, 11 of them loaded substantially on only the general factor. Moreover, all of the remaining nine items had a higher loading on the general factor than the specific factor.

Finally, age and gender were added into the bi-factor model as a MIMIC model. The MIMIC model fits the data acceptably well and showed two substantive direct effects from gender to two items. Being female was negatively associated [*β* = −0.42, standard error (SE) = 0.08, *p* < .01] with item 13 (talked less than usual) and positively associated (*β* = 0.64, SE = 0.10, *p* < .01) with item 17 (crying spells). Controlling for the direct effects, there was no significant gender difference in the general factor (*β* = 0.14, SE = 0.09, *p* > .05), the positive affect factor (*β* = –0.18, SE = 0.10, *p* > .05), or the interpersonal problems factor (*β* = −0.12, SE = 0.12, *p* > .05). One exception was that women had significantly lower scores in the somatic symptoms factor (*β* = –0.33, SE = 0.12, *p* < .01). Age was found to be negatively associated with the general factor (*β* = −0.09, SE = 0.03, *p* < .05) but not with the three specific factors (*p* > .05).

## Discussion

The present study evaluated the dimensionality of the CES-D scale via two sets of measurement models: the commonly used CFA models and the new exploratory bi-factor models. The single-factor CFA model showed a mediocre fit. The poor model fit could be attributed to violations of conditional independence assumptions. Because of the diverse item contents of the CES-D, the items are seldom strictly unidimensional. Consistent with previous research [2, 3, 6, 9], the four-factor model fitted the data significantly better than the single-factor model. However, the strong inter-factor correlations (*r* > .6) appear to suggest substantial overlapping among the dimensions and potential model redundancy. The second-order factor model that explained the high correlations resulted in Heywood cases, implying misspecification for the second-order factor. Overall, the CFA results failed to support any of the existing measurement models of the CES-D.

The exploratory bi-factor model results showed a dominant general factor that accounted for more than half of the total item variance. All items had a higher loading on the general factor than on the specific factors, with more than half of them loading substantially only on the general factor. In comparison, the specific factors showed weak factor loadings and provided little unique information over and above the general factor, implying that the specific factors might not be well measured by the items. The specific factor for positive affect comprised the four positively worded items and could plausibly represent a methodological artifact rather than a substantive specific factor. Similarly, the specific factor for interpersonal problems could denote residual item covariation and could rather be replaced by a correlated error.

The present findings suggest greater measurement precision for the general factor and that the bi-factor model may provide a better representation of the underlying structure. Overall, these results support an argument that the CES-D is an approximately unidimensional measure, and the use of the CES-D general factor as a screening measure of depressive symptoms is justified. Bi-factor modeling offers a useful alternative to traditional multidimensional models and can provide new insights into dimensionality assessment [21]. The bi-factor model deals effectively with violations of local independence caused by item clustering via specific factors, allows the separation of item variance into general and specific components, and enables researchers to evaluate the utility of the specific factors [17, 32].

The general depression factor was found to be negatively associated with age, which was generally consistent with previous research [2–6]. The current sample did not show gender differences in the overall level of depressive symptoms, and most of the CES-D items showed no gender bias. Differential item functioning across the genders was found for item 13 (talked less than usual) and item 17 (crying spells). The measurement bias possibly reflects that women tend to be more sociable and emotionally expressive than men and are thus less likely to endorse item 13 but more likely to endorse item 17 than men regardless of their depression level. To avoid potential measurement bias across gender, future studies might consider excluding these two items from the scale.

A limitation of this study is that the current sample was based on moderately depressed persons who voluntarily enrolled in the trial of qigong and body–mind–spirit interventions. The current findings may not generalize to the patient population with different severities of depressive symptoms. Future studies could investigate the suitability of the bi-factor model in identifying depressive symptoms and examine its measurement invariance across varying degrees of psychopathology in large statistically representative clinical samples. The present results are based only on self-reported cross-sectional data. Longitudinal studies are needed to evaluate the stability and changes in the general and specific factors over time. Item 11 (restless sleep) showed a rather low loading (*λ* = .43) on the general factor. This finding could be attributed to the fact that over 60 % of the participants reported sleep disturbance most of the time and the associated low interindividual variation. Further research is encouraged to elucidate the comorbid nature between sleep disturbance and depressive symptoms.

In conclusion, this psychometric study was the first to explore the bi-factor model to evaluate the dimensionality of the CES-D for a unique sample of Chinese adults. The present study demonstrated empirical support for the bi-factor model as a useful and realistic representation of the underlying structure. Future studies could explore the predictive validity of the general and specific factors on external variables. In particular, the bi-factor model allows assessment of the unique contribution of specific factors to prediction after controlling for the general factor. Rather than a multidimensional scoring system, it is recommended that researchers and clinicians use the CES-D total score as a precise and parsimonious assessment of depressive symptoms.
